# Long-lasting semantic interference effects in object naming are *not* necessarily conceptually mediated

**DOI:** 10.3389/fpsyg.2015.00578

**Published:** 2015-05-07

**Authors:** Emma Riley, Katie L. McMahon, Greig de Zubicaray

**Affiliations:** ^1^School of Psychology, University of QueenslandBrisbane, QLD, Australia; ^2^Centre for Advanced Imaging, University of QueenslandBrisbane, QLD, Australia

**Keywords:** language production, lexical retrieval, semantic interference

## Abstract

Long-lasting interference effects in picture naming are induced when objects are presented in categorically related contexts in both continuous and blocked cyclic paradigms. Less consistent context effects have been reported when the task is changed to semantic classification. Experiment 1 confirmed the recent finding of cumulative facilitation in the continuous paradigm with living/non-living superordinate categorization. To avoid a potential confound involving participants responding with the identical superordinate category in related contexts in the blocked cyclic paradigm, we devised a novel set of categorically related objects that also varied in terms of relative age – a core semantic type associated with the adjective word class across languages. Experiment 2 demonstrated the typical interference effect with these stimuli in basic level naming. In Experiment 3, using the identical blocked cyclic paradigm, we failed to observe semantic context effects when the same pictures were classified as younger–older. Overall, the results indicate the semantic context effects in the two paradigms do not share a common origin, with the effect in the continuous paradigm arising at the level of conceptual representations or in conceptual-to-lexical connections while the effect in the blocked cyclic paradigm most likely originates at a lexical level of representation. The implications of these findings for current accounts of long-lasting interference effects in spoken word production are discussed.

## Introduction

The mechanism by which words are retrieved from the mental lexicon for production remains a topic of considerable debate. One of the principal methods for investigating this mechanism in healthy participants involves the use of picture naming paradigms. The speeds at which pictures can be named can be altered by experimental manipulations of context variables. For example, categorically related compared to unrelated contexts typically induce interference effects in picture naming paradigms. These interference effects can also be relatively persistent, surviving multiple intervening unrelated trials. This paper is concerned with identifying the origin(s) of long-lasting semantic context effects in two well-established experimental word production paradigms: continuous and blocked cyclic picture naming. Findings with both paradigms have been integral to the development of rival theoretical accounts of spoken word production (Howard et al., 2006; Oppenheim et al., 2010; Belke, 2013). It is therefore important to establish whether the two different manipulations of semantic context involve identical or different mechanisms.

The continuous paradigm requires participants to name multiple exemplars (e.g., 5) from a large set of categories (e.g., 24), each separated by varying numbers of intervening, unrelated objects (i.e., lags). Importantly, participants are *not* familiarized with the objects and none are repeated. The typical finding with this paradigm is a cumulative inhibition (or interference) effect: naming latencies increase monotonically with each ordinal presentation within a category, unaffected by lags of up to eight items (Howard et al., 2006; Navarrete et al., 2010; Oppenheim et al., 2010; Runnqvist et al., 2012; Belke, 2013; Belke and Stielow, 2013; Kleinman, 2013; Schnur, 2014). By contrast, the blocked cyclic paradigm entails small sets of category exemplars (e.g., 4–6) presented repeatedly over several cycles (e.g., 4–6) in alternating and contrasting contexts. Additionally, participants are first familiarized with the objects to be named. Within homogeneous contexts, objects are exemplars of the same category (e.g., all animals), while heterogeneous blocks comprise exemplars from different categories (e.g., animal, vehicle, furniture, fruit). Participants are slower to name objects in homogeneous compared to heterogeneous blocks (Kroll and Stewart, 1994; Damian et al., 2001; Damian and Als, 2005; Abdel Rahman and Melinger, 2007). This interference effect usually manifests from the second presentation cycle onward (Belke and Stielow, 2013).

The interference effects in both paradigms have generally been explained in terms of operations involving conceptual and/or lexical levels of representation. All models of spoken word production assume these representational levels. However, in order to explain cumulative and long-lasting interference in naming, it has been argued that these models need to incorporate additional priming mechanisms (Wheeldon and Monsell, 1994; Damian and Als, 2005; Howard et al., 2006). For example, Howard et al. (2006) proposed three essential mechanisms to explain the cumulative interference effect observed in continuous naming. The first is *shared activation of conceptual features* among categorically related objects. The second is *repetition priming* of conceptual-to-lexical connections or lexical representations, while the third mechanism is *lexical selection by competition*. According to this account, the interference effect in continuous naming arises due to strengthening of conceptual-to-lexical connections among existing candidates for selection and therefore has a lexical locus.

Oppenheim et al. (2010) have also proposed the context effect in continuous naming arises in conceptual-to-lexical connections. However, priming in their model is implemented as an incremental learning mechanism (e.g., Damian and Als, 2005) that both strengthens conceptual-to-lexical connections for targets and weakens connections for competing lexical candidates as target items are named, making those representations less accessible on later trials. In addition, lexical selection is accomplished by a threshold mechanism (i.e., the most highly activated word is retrieved, regardless of the activation levels of non-target words) rather than competitive selection mechanism (in which the activation levels of non-target candidates also influence selection). Using both of these mechanisms, Oppenheim et al. (2010) were able to simulate the interference effects in both continuous and blocked cyclic naming paradigms. Navarrete et al. (2010) have similarly proposed that the interference effect in continuous naming does not involve competitive lexical selection.

There is little consensus about either the origin or locus of the semantic interference effect in the blocked cyclic paradigm, with some authors proposing it arises due to residual activation accumulating in conceptual representations leading to greater lexical competition (Kroll and Stewart, 1994; Belke, 2013), or that it originates in conceptual-to-lexical connections and has a lexical locus (Damian and Als, 2005; Oppenheim et al., 2010; Navarrete et al., 2012) or that it both originates and has its locus at the lexical level (Damian et al., 2001). Establishing the origin and locus of semantic context effects in picture naming paradigms is important as it can provide evidence to support or refute rival production models. For instance, if it can be shown that the origin of a semantic context effect in naming is at a conceptual level of processing yet its locus is post-lexical, then a lexical selection by competition model can be refuted (see Navarrete et al., 2010, 2012, 2014). In addition, if the origins of semantic interference effects across paradigms can be shown to differ, then accounts assuming a common origin for the effects can be refuted (e.g., Oppenheim et al., 2010; Belke, 2013).

In order to determine if the semantic interference effect in the blocked cyclic paradigm arises at a conceptual processing level, Damian et al. (2001) conducted an experiment involving orientation judgments, i.e., a decision with respect to the direction in which an object typically faces. For example, all animals ‘face’ toward their heads, irrespective of whether they are depicted pointing in a left or right direction, allowing responses to be counterbalanced. Thus, feature sharing should result in converging activation within a block of categorically related items. Damian et al. (2001) proposed that if the origin and locus of the interference effect in blocked cyclic naming were at the conceptual level, then an effect should also be observed with orientation judgments. However, they failed to find a context effect. They concluded that the semantic interference effect observed with basic level naming therefore had both a lexical origin and locus, interpreting their results according to a competitive selection account (Levelt et al., 1999).

Over a series of experiments with both continuous and modified blocked cyclic paradigms, Belke (2013) recently observed consistent facilitatory effects of semantic context on superordinate (living/manmade) classifications, concluding that the context effects in both paradigms arise due to residual activation accumulating in conceptual representations (e.g., Kroll and Stewart, 1994). In order to explain the discrepant results for semantic classification and orientation judgments in the blocked cyclic paradigm, Belke proposed Damian et al. (2001, p. 230) use of orientation judgments “requires access to structural object descriptions only and does not necessarily involve conceptual processing.” However, this proposal is not consistent with the findings of Boucart and Humphreys (1994, 1997). In two multi-experiment studies using a successive object-matching task, they observed consistent context effects when participants matched the orientations of targets to reference objects in the presence of categorically related vs. unrelated distractors: performance was facilitated when the target and reference objects were categorically related, and an interference effect was observed when the distractor was categorically related to the reference object compared to an unrelated object. They argued that responses to categorically related targets are facilitated due to feature overlap between reference and target, while the distractor interference effect occurs because selection is more difficult when reference, target and distractor belong to the same category (i.e., a competitive selection mechanism). Boucart and Humphreys (1994, 1997) concluded that participants were unable to prevent automatic access to conceptual information when making orientation judgments.

The discrepancy between Damian et al.’s (2001) and Belke’s (2013) results might instead be due to the use of a semantic classification task in conjunction with a highly modified procedure. In classification experiments, participants are usually instructed to respond verbally to a given object with its superordinate-level category name (Lupker and Katz, 1981; Glaser and Düngelhoff, 1984; Glaser and Glaser, 1989; Humphreys et al., 1995; Costa et al., 2003; Hantsch et al., 2012), although manual classification has also occasionally been employed (Damian et al., 2001; Belke, 2013). Superordinate categories are the most general, incorporating more abstract information about objects (e.g., whether they are living or non-living). Context effects in semantic classification have been attributed to activation of shared conceptual features converging on the *same* response, resulting in facilitation, rather than spreading to multiple lexical candidates, as is proposed in the case of basic-level naming (Lupker and Katz, 1981; Glaser and Düngelhoff, 1984; Glaser and Glaser, 1989; Humphreys et al., 1995; Costa et al., 2003; Kuipers and La Heij, 2008; Hantsch et al., 2012). However, the use of superordinate (living/non-living) categories introduces a confound in the blocked cyclic paradigm due to participants responding with the identical category on consecutive trials in homogeneous contexts (e.g., all living) and alternating living and non-living responses to items in heterogeneous contexts. Damian et al.’s (2001) use of orientation judgments avoided this response confound while maintaining the blocked cyclic procedure.

Of note, Belke’s (2013) study entailed procedural modifications to both paradigms. The modification to the continuous paradigm involved first familiarizing participants with the objects to be named (cf., Howard et al., 2006). The blocked cyclic paradigm was modified by adding a lag manipulation with unrelated filler trials to avoid identical consecutive responses to items in homogeneous contexts, creating ‘supersets’ of items. Typically, semantic interference effects in picture naming manifest from the second presentation cycle in the blocking paradigm (see Belke and Stielow, 2013). However, Damian and Als (2005; Experiments 1, 3, 4A,B) showed that the addition of unrelated filler trials yielded a different outcome, with the interference effect now observable from the first cycle. This was also the case in Belke’s (2013) Experiment 2. Damian and Als (2005) proposed the addition of filler trials eliminated a short-lived facilitation or self-inhibitory effect that also operated in the paradigm (see Navarrete et al., 2012 for a similar proposal; e.g., Wheeldon and Monsell, 1994; Vitkovitch et al., 2001).

Belke and Stielow (2013) have shown that a concurrent digit-retention task exacerbates the context effect in the conventional blocking paradigm yet does not influence the context effect in the continuous paradigm. Crowther and Martin (2014) have also shown the interference effect in the blocking paradigm is correlated with a measure of short term memory span. According to Belke and Stielow (2013), the “crucial difference” between the continuous and conventional blocked cyclic paradigms is that participants are able to distinguish task-relevant from task-irrelevant representations in the latter paradigm, as they are able to memorize the task set as of the first presentation cycle onward. Thus, they concluded working memory plays a “selective role” in the blocked cyclic paradigm. Yet, modifying the blocking paradigm with the lag manipulation ensured “there were no discernible cycles and the members of the homogeneous and the heterogeneous set mixed in one list appeared in an unpredictable order” (Belke, 2013, p. 237).

Eliminating an obvious task set in the blocked cyclic paradigm by adding a lag manipulation might also make it more similar to the continuous paradigm, leading to the conclusion that context effects in both paradigms share a common origin. For example, Navarrete et al. (2010; Experiment 1) had participants perform the continuous naming paradigm four times and reported a significant main effect of repetition, yet no significant interaction with the context effect. Using the modified blocked cyclic naming paradigm, both Damian and Als (2005; Experiment 4B) and Belke (2013) reported significant main effects of repetition/cycle yet no interaction with semantic context, a finding that differs from the significant interaction typically reported with the conventional version (see Belke and Stielow, 2013). Note that merely adding a lag manipulation to the blocked cyclic paradigm would be unlikely to produce a cumulative interference effect like the one observed in the continuous paradigm, as Howard et al. (2006) showed the cumulative effect was not influenced by lags of up to eight items, a finding that has been replicated consistently (see Schnur, 2014). Neither Damian and Als (2005; Experiment 4B) nor Belke (2013; Experiment 2) observed a cumulative interference effect with the modified blocking paradigm, and Belke (2013) also failed to observe a significant main effect of lag or interaction of lag with context in follow-up analyses.

### The Present Study

The purpose of the present study was to test whether the semantic context effects in both continuous and blocked cyclic paradigms have identical or different origins. We therefore employed a verbal semantic classification task. In Experiment 1, using the unmodified continuous paradigm (i.e., without a picture familiarization phase), our aim was to replicate Belke (2013; Experiment 1) finding of cumulative facilitation using living/non-living judgments. All current accounts of the context effect in that paradigm hypothesize that it arises in conceptual representations or in conceptual-to-lexical connections (Howard et al., 2006; Navarrete et al., 2010; Oppenheim et al., 2010; Belke, 2013). As an interference effect has been replicated multiple times in that paradigm with basic level naming (Costa et al., 2009; Navarrete et al., 2010; Oppenheim et al., 2010; Runnqvist et al., 2012; Belke, 2013; Belke and Stielow, 2013; de Zubicaray et al., 2013; Kleinman, 2013; Schnur, 2014), we tested only superordinate categorization.

Modifying the blocked cyclic paradigm with a lag manipulation to prevent participants responding with the identical superordinate category on consecutive trials in homogeneous contexts might render it more similar to the continuous paradigm. It is also possible that orientation judgments might not necessarily involve accessing conceptual features (but see Boucart and Humphreys, 1994, 1997). We therefore devised a novel set of categorically related object stimuli that also varied systematically in terms of *relative age*, permitting alternating category responses to be made in both homogeneous and heterogeneous contexts without the need of a lag modification. Across languages, the relative age dimension (i.e., *younger–older*) is considered a core semantic type associated with the adjectival word class (Justeson and Katz, 1995; Dixon and Aikhenvald, 2004). According to linguists, the meanings of relative adjectives (e.g., *old, tall, cheap*) are defined according to a relevant comparison class (or scale), with the speaker assigning a reference point (i.e., a *norm*) around the middle of the scale. Importantly, a comparison class is usually determined by the immediate context. However, it can also be supplied from a broader linguistic and/or non-linguistic context (e.g., encyclopedic knowledge; see Tribushinina, 2011).

Psycholinguists have a long history of demonstrating semantic context effects using relative adjective dimensions. The *semantic congruity* effect refers to the finding that participants are faster to compare objects within an experimental series when the direction of comparison is congruent with the objects’ position on a relevant dimension (e.g., Shipley et al., 1945; Ellis, 1972; Banks et al., 1975; Ryalls and Smith, 2000). For example, when instructed to make judgments about the sizes of animals, participants are typically faster at choosing the larger of two relatively large animals (e.g., *elephant* vs. *hippopotamus*) than at choosing the smaller of two relatively large animals. They are also faster when choosing the smaller of two relatively small animals (e.g., *mouse* vs. *guinea pig*).

Semantic congruity effects have been demonstrated for a range of relative adjective dimensions (e.g., size, color, brightness; Shipley et al., 1945; Banks et al., 1975) as well as for artificial dimensional adjectives, indicating participants are able to create and use lexical-semantic categories “on the fly” (e.g., Ryalls and Smith, 2000). Importantly, the semantic congruity effect is context-specific; when the range of stimuli is altered within a single experiment the effect “adapts” to the new continuum (e.g., Cech and Shoben, 1985). The dominant explanation of the effect is that participants’ first process the conceptual features of an object series according to the task instructions, and then assign them around the midpoint of a context-specific comparison class in order to make a comparative judgment (see Ryalls and Smith, 2000). Ellis (1972) was the first to demonstrate a semantic congruity effect for the younger–older adjectival dimension.

As the blocked cyclic paradigm involves presenting objects in categorically related contexts, older–younger judgments should therefore show a context effect due to feature overlap if the interference effect in basic level naming arises at the conceptual level. In Experiment 2, we first establish that context objects varying according to relative age induce the semantic interference effect observed typically for basic-level naming in the blocked cyclic paradigm. In Experiment 3, the same paradigm was employed and the task was changed to categorization with younger-older judgments. Belke (2013, p. 230) provides a concise explanation of the logic for employing superordinate categorization: “Critically, the prediction is that *any* task that encompasses the level of processing deemed to be the origin of the semantic context effect should yield context effects; conversely, tasks that do not yield semantic context effects are diagnostic of the levels of processing that cannot be deemed as the origin of the semantic context effect” (emphasis added). Thus, if the semantic context effects in continuous and blocked cyclic paradigms have a common origin in conceptual processing, then they should be observed with superordinate categorization in both paradigms, irrespective of the nature of the semantic classification employed (i.e., living/non-living, orientation or younger-older).

## Experiment 1: Continuous Paradigm with Superordinate Categorisation

### Method

#### Participants

Twenty-four students enrolled in a first year psychology course at the University of Queensland completed the experiment in exchange for partial course credit. All participants reported normal or corrected-to-normal vision, and no history of neurological or psychiatric disorder, or substance dependence. All participants identified as right-handed, native English speakers. All participants gave written informed consent in accordance with the experimental protocol approved by the Behavioural and Social Sciences Ethical Review Committee (BSSERC) of the University of Queensland.

#### Materials and Procedure

**FIGURE 1:**
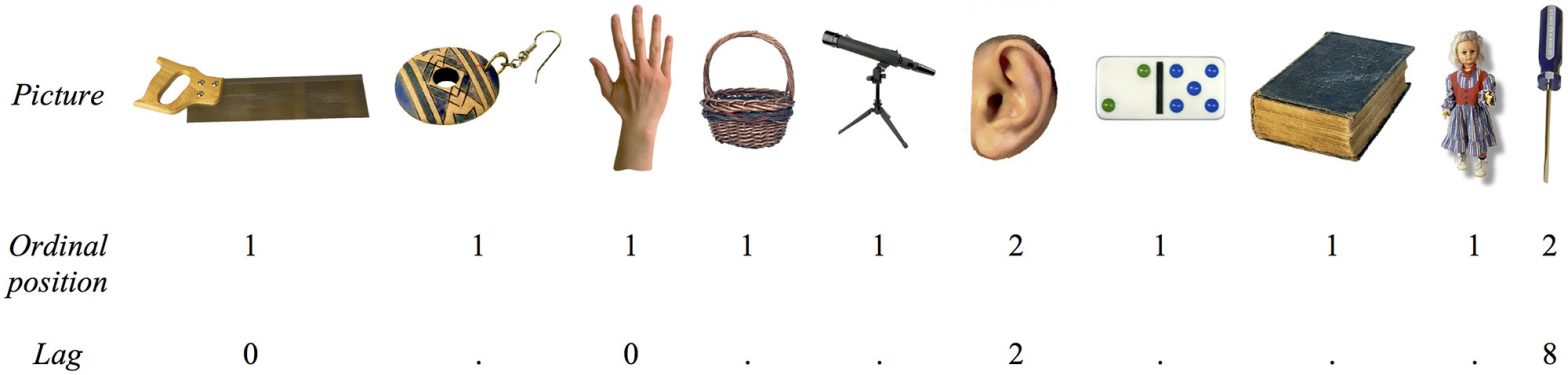
**An example sequence of consecutive trials in the continuous paradigm employed in Experiment 1.** *Saw* and *screwdriver* are members of the same category of “tools” in ordinal positions 1 and 2, respectively, and separated by a lag of eight items, whereas *hand* and *ear* are members of the same category “body parts” separated by a lag of two items. The remaining items are fillers.

The materials (pictures and experimental lists) for the continuous paradigm were identical to those employed by Howard et al. (2006). The pictures comprised 165 color photographs; 120 of which were experimental target items comprising five exemplars from each of 24 categories. The remaining 45 pictures were filler items unrelated to the targets. Of the experimental items, 10 of the 24 categories were living things (i.e., 70 items were non-living objects). Forty of the filler items were likewise non-living objects. This imbalance between living and non-living items was unavoidable, due to the way in which Howard et al. (2006) created their experimental lists to randomize both lags and ordinal positions of their items, with the constraint that targets and fillers should be semantically unrelated. Howard et al. (2006, p. 468) adopted this procedure to both minimize participants’ awareness that specific semantic categories were being repeatedly probed, and to avoid any short-term effects of naming items selected from one semantic category. However, as Belke (2013) noted, although this imbalance might result in a response bias affecting superordinate categorisation latencies between living and non-living categories, it would not necessarily be expected to influence within-category responses. In each of 24 experimental lists (each corresponding to a separate participant), category exemplars were separated by 2, 4, 6, or 8 intervening items (i.e., lags), and each lag order was realized equally often with each category (i.e., once). In addition, each category had a different ordering of lag. An example of a sequence of consecutive trials with experimental and filler items in the continuous paradigm is shown in **Figure 1**.

The experiment was run with the Cogent 2000 toolbox (version 1.32^1^) in MATLAB (Version 7.13, R2011b, The Mathworks; Natick, MA, USA). Pictures were displayed centrally in 32 colors on a white background. Each trial commenced with a fixation cross for 500 ms, followed by a blank screen for 250 ms. A picture was then presented for 2000 ms, and was followed by a blank screen for 500 ms. Response times (RTs) were recorded by a voice key implemented in Cogent 2000. Participants were instructed to categorize each picture as either living or non-living, as quickly and as accurately as possible.

### Results

All filler items were excluded from analyses. Technical errors (in which the voice-key failed to detect a response) accounted for 0.28% of items being excluded. Outliers were dealt with in an identical manner to Howard et al. (2006) and Belke (2013): vocal responses faster than 250 ms (1.01%) and slower than 2000 ms (0.10%) from picture onset were excluded. Classification errors were infrequent (4.71%) and so were not subjected to further analysis.

We conducted two separate repeated measures analyses of variance (ANOVAs) each with participants (*F*_1_) and categories (*F*_2_) as random factors. The first analysis was restricted to items in ordinal positions two to five to examine the effect on superordinate categorisation latencies of ordinal position with a category, and the effect of the last presentation of an item within a category (i.e., the effect of lag). The second analysis was conducted to investigate the effects of ordinal position within a category on latencies. This analysis included data from all five ordinal positions, and collapsed across lag. Although Howard et al.’s (2006) design minimized the potential confound between ordinal position with category and serial position, it did not eliminate it. Therefore, we adjusted participant’s classification times for any linear trends over the experiment following Howard et al. (2006). The effect of serial position was not significant, by participants or categories (*F*_1_ and *F*_2_ both <1, *p* > 0.05). Thus, the following analyses are based on unadjusted reaction times per Howard et al. (2006) and Belke (2013). The adjusted and unadjusted reaction times are presented in **Table 1**.

**Table 1:** Mean correct picture categorization latencies (in milliseconds) as a function of ordinal position and lag in the continuous paradigm (Experiment 1).

	Ordinal position					
Lag	1	2	3	4	5	Mean
**A**
2		686.69 (12.21)	668.99 (12.56)	647.39 (12.19)	656.16 (12.19)	664.81
4		681.22 (12.33)	672.84 (12.19)	662.47 (12.47)	642.17 (11.95)	664.68
6		667.19 (12.33)	665.72 (12.12)	642.13 (12.19)	663.18 (12.28)	659.56
8		685.15 (12.48)	664.81 (12.25)	653.31 (12.21)	667.92 (12.38)	667.80
**Mean**	696.62	680.06	668.09	651.34	657.36	
**B**
2		699.38 (13.37)	688.61 (13.38)	656.16 (13.33)	663.72 (13.40)	676.97
4		693.92 (13.38)	684.37 (13.22)	672.11 (13.24)	647.60 (13.19)	674.50
6		669.69 (13.47)	679.48 (13.17)	647.23 (13.29)	675.34 (13.24)	667.94
8		706.01 (13.24)	676.47 (13.29)	654.55 (13.28)	676.64 (13.35)	678.42
**Mean**	709.60	692.25	682.23	657.51	665.83	

A, unadjusted latencies; B, latencies adjusted for linear changes over the experiment. Standard errors are in parentheses.

**FIGURE 2:**
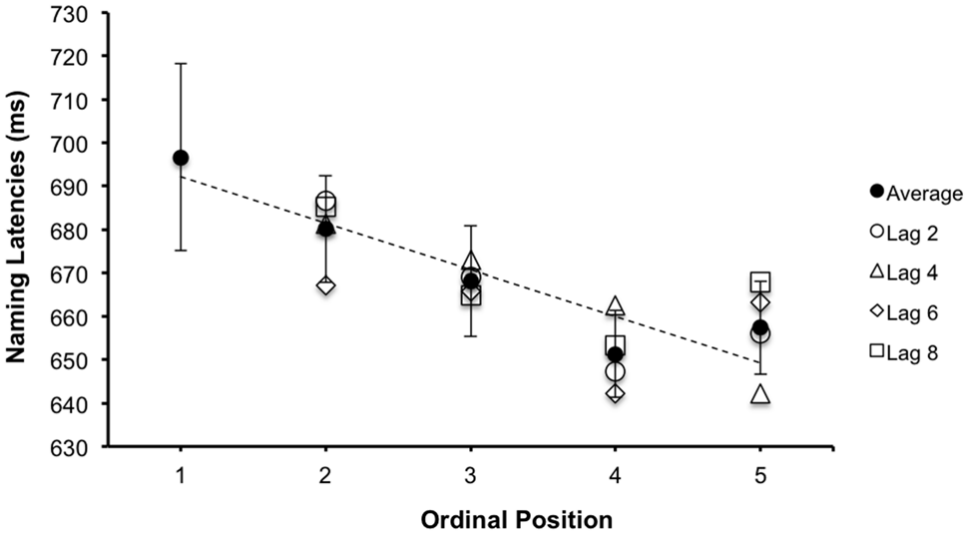
**Mean correct superordinate categorization latencies as a function of ordinal position and lag in the continuous paradigm.** Error bars represent 95% confidence intervals (CIs) calculated per Cousineau’s (2005) method.

For the first analysis restricted to items in ordinal positions two to five, there was a significant main effect of ordinal position [*F*_1_ (3,69) = 3.23, MSE = 22913.58, *p* = 0.025, $\eta_{p}^{2}$ = 0.073; *F*_2_(3,69) = 5.45, MSE = 19915.91, *p* = 0.002, $\eta_{p}^{2}$ = 0.184]. However, there was no effect of lag [*F*_1_(3,69) = 0.25, MSE = 19035.59, *p* = 0.911, $\eta_{p}^{2}$ = 0.008; *F*_2_(3,69) = 0.29, MSE = 18932.92, *p* = 0.887, $\eta_{p}^{2}$ = 0.007], or interaction between ordinal position and lag [*F*_1_(3,69) = 0.52, MSE = 19483.44, *p* = 0.874, $\eta_{p}^{2}$ = 0.020; *F*_2_(3,69) = 0.72, MSE = 23942.56, *p* = 0.689, $\eta_{p}^{2}$ = 0.092]. The second analysis, which included data from all five ordinal positions, confirmed the significant main effect of ordinal position [*F*_1_(4,92) = 7.19, MSE = 24796.93, *p* ≤ 0.001, $\eta_{p}^{2}$ = 0.237; *F*_2_(4,92) = 10.45, MSE = 20443.96, *p* ≤ 0.001, $\eta_{p}^{2}$ = 0.305]. A significant linear trend of this effect was found via a contrast of coefficients corresponding to a first order polynomial [*F*_1_(1,23) = 23.01, MSE = 1210.37, *p* < 0.001, $\eta_{p}^{2}$ = 0.500; *F*_2_ (1,23) = 28.06, MSE = 33494.34, *p* < 0.001, $\eta_{p}^{2}$ = 0.550]. As shown in **Figure 2**, there is a monotonic decrease in classification times from ordinal position 1–4. However, there is no further decrease. Interestingly, we also found a non-significant trend by participants for a quadratic effect via a contrast of coefficients corresponding to a second order polynomial [*F*_1_(1,23) = 2.28, MSE = 1217.86, *p* = 0.144, $\eta_{p}^{2}$ = 0.090] that was significant by categories [*F*_2_(1,23) = 6.22, MSE = 2997.45, *p* = 0.020, $\eta_{p}^{2}$ = 0.213], reflecting a slight increase in classification times (∼6 ms) between ordinal positions 4 and 5.

Following Belke (2013), we next analyzed superordinate categorisation times according to response type (living/non-living) and ordinal position. As the data in **Table 2** show, the mean RTs for the living and non-living items showed similar patterns over the five ordinal positions. There were significant main effects of ordinal position [F_1_(4,92) = 6.11, MSE = 26070.68, *p* < 0.001, $\eta_{p}^{2}$ = 0.208; *F*_2_(4,92) = 23.96, MSE = 7090.56, *p* = 0.005, $\eta_{p}^{2}$ = 0.960], and response type [*F*_1_(1,92) = 7.88, MSE = 32442.46, *p* = 0.010, $\eta_{p}^{2}$ = 0.253; *F*_2_(1,92) = 49.05, MSE = 7092.01, *p* = 0.002, $\eta_{p}^{2}$ = 0.925]. However, the interaction was not significant [*F*_1_ and *F*_2_ both <1, *p* > 0.05]. Separate trend analyses of each response type revealed significant linear effects for both living [*F*_1_(1,23) = 6.58, MSE = 2552.22, *p* = 0.017, $\eta_{p}^{2}$ = 0.223; *F*_2_(1,9) = 5.471, MSE = 11011.656, *p* = 0.044, $\eta_{p}^{2}$ = 0.378] and non-living items [*F*_1_(1,23) = 21.80, MSE = 1743.87, *p* < 0.001, $\eta_{p}^{2}$ = 0.49; *F*_2_(1,13) = 32.769, MSE = 22781.338, *p* < 0.001, $\eta_{p}^{2}$ = 0.716]. A significant quadratic effect was also discernible for non-living items [*F*_1_(1,23) = 5.04, MSE = 986.48, *p* = 0.035, $\eta_{p}^{2}$ = 0.180; *F*_2_(1,13) = 11.659, MSE = 2267.637, *p* = 0.005, $\eta_{p}^{2}$ = 0.473], while the quadratic effect was not significant for living items [*F*_1_ and *F*_2_ both <1, *p* > 0.05].

**Table 2:** Mean correct picture categorization latencies (in milliseconds) as a function of ordinal position and response type in the continuous paradigm (Experiment 1).

	Ordinal Position					
Category	1	2	3	4	5	Mean
Living	705.10 (11.67)	693.74 (11.61)	688.72 (11.59)	666.17 (11.45)	674.49 (11.45)	685.44
Non-living	693.42 (9.503)	672.90 (9.55)	657.98 (9.49)	641.48 (9.54)	646.51 (9.47)	662.46
**Mean**	698.08	681.31	670.32	651.60	657.88	671.74

Standard errors are in parentheses.

As Alario and Moscoso del Prado Martin (2010) noted, one shortcoming of the above analyses is the circular approach taken to address the confound between ordinal and trial positions. This is because adjusting for a linear trend over trials involves estimating and correcting for the effect of trial position by using the same data (e.g., Howard et al., 2006; Belke, 2013). Alario and Moscoso del Prado Martin (2010) therefore recommended the use of mixed-effect modeling for the continuous naming paradigm (e.g., Baayen et al., 2008). This is potentially more problematic for superordinate classification than basic level naming due to the reduced response variability, making an increase in classification efficiency more likely over the course of the experiment. The advantage of mixed effect analysis is that it allows the contributions of the different ordinal and trial position factors to be characterized at the single trial level.

We submitted the classification latencies to mixed effects analysis, after first log transforming them to reduce skewness and approach a normal distribution (Alario and Moscoso del Prado Martin, 2010). Ordinal position, lag and trial number were entered as fixed effects, while participant, picture/item and superordinate category response type were included as random effects. We also included interactions between the fixed effect of trial number and the random effect of participants and between the fixed effect of ordinal position and random effect of superordinate category (i.e., mixed effects). The model was estimated using Restricted Maximum Likelihood (ReML). As **Table 3** shows, there was a significant facilitatory effect of ordinal position on classification latencies, but no significant effects of lag, trial number or superordinate category.

**Table 3:** Comparison of the fixed and random effects in linear-mixed modeling of the log-transformed picture categorization latencies (Experiment 1).

Parameter	Estimate	*t*	*p*
Intercept	6.55	145.97	<0.001
Ordinal position	-0.027	-2.74	=0.006
Lag	0.0013	0.2	=0.85
Trial number	0.0007	1.68	=0.09
Superordinate Category	0.008	2.12	=0.17

### Discussion

We observed a significant semantic context effect on superordinate categorisation latencies in the continuous paradigm. This effect, significant in both the conventional ANOVA and linear mixed effects analyses, manifested as cumulative facilitation, with categorisation of each picture speeded when preceded by a semantically related item. This supports the findings for button-press semantic classification reported by Belke (2013; Experiment 1) using a within-participants design that additionally included a familiarization phase and concurrent tasks. We also observed significant effects according to response type in the conventional ANOVA, indicating non-living items were categorized more quickly (∼20 ms) than living items overall, consistent with Belke’s (2013) findings. However, the effect of response type was not significant in the linear mixed effects model, suggesting the findings with the conventional ANOVA approach might be due to averaging of data across items or participants, and so are not reliable. The finding of a significant context effect with superordinate categorisation is consistent with an origin in conceptual representations (Kroll and Stewart, 1994; Belke, 2013) or in conceptual-to-lexical connections (Howard et al., 2006; Oppenheim et al., 2010).

## Experiment 2: Blocked Cyclic Paradigm with Basic Level Naming

The purpose of Experiment 2 is to determine whether a typical semantic context effect can be elicited in the blocked cyclic paradigm with basic-level naming by a set of novel, categorically related items that additionally vary in terms of the superordinate semantic category of *relative age* (Ellis, 1972). This demonstration is necessary before conducting an experiment employing relative age judgments in the blocking paradigm to test the hypothesis of a conceptual vs. lexical locus for the context effect. The relative age manipulation is designed to avoid the response confound in superordinate categorization introduced by blocking categorically related objects and to preserve the context manipulation across basic naming and superordinate categorization tasks (see Belke, 2013; e.g., Damian et al., 2001). However, before proceeding to picture categorization, it is first necessary to demonstrate that the novel set of stimuli induce the typical semantic interference effect in object naming.

### Method

#### Participants

Sixteen students enrolled in a first year psychology course at the University of Queensland completed the experiment in exchange for partial course credit. None had participated in Experiment 1. All participants reported normal or corrected-to-normal vision, and no history of neurological or psychiatric disorder, or substance dependence. All participants identified as right-handed, native English speakers. All participants gave written informed consent in accordance with the experimental protocol approved by the Behavioural and Social Sciences Ethical Review Committee (BSSERC) of the University of Queensland.

#### Materials and Procedure

**FIGURE 3:**
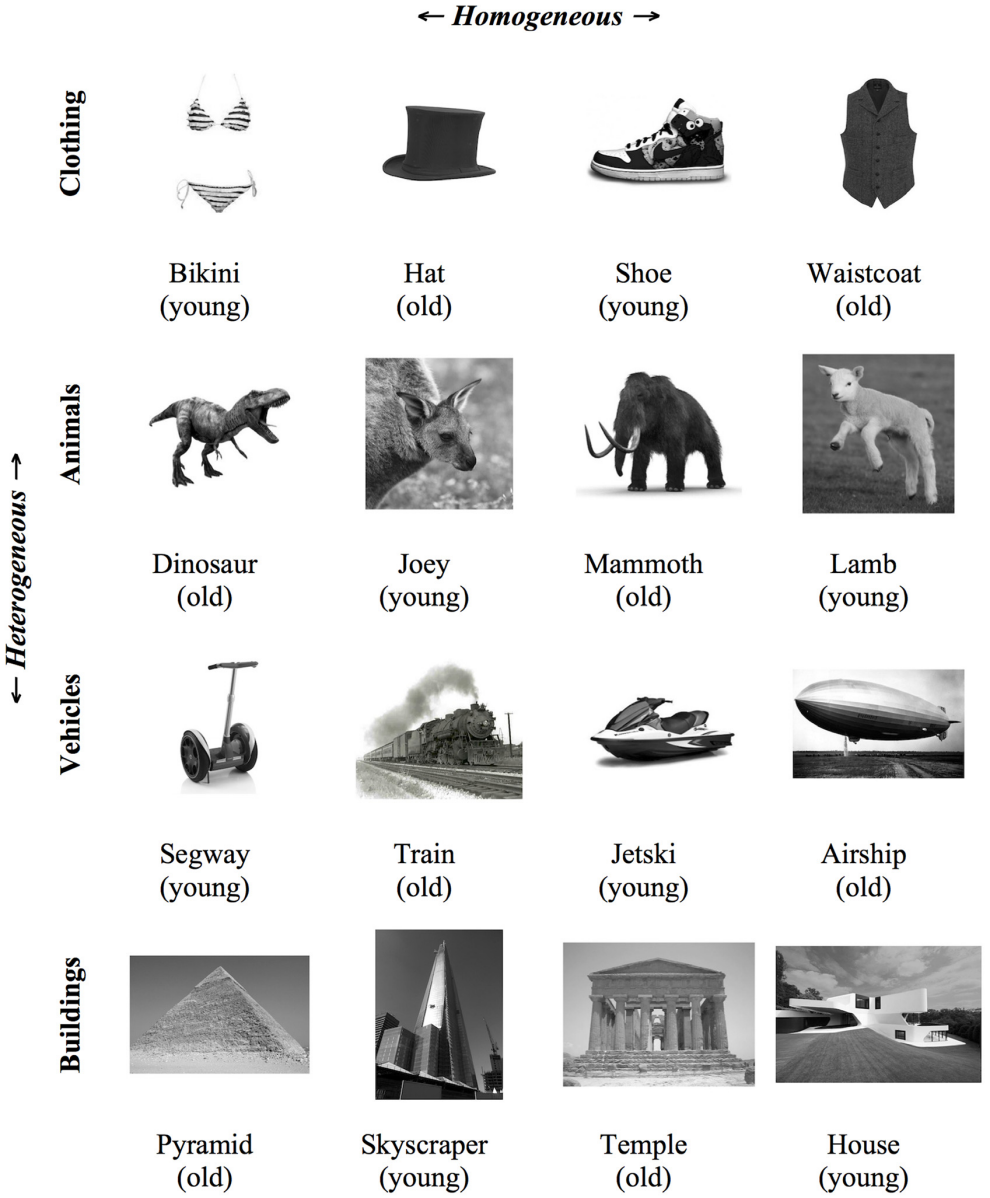
**Matrix depicting the novel object stimuli employed in Experiments 2 and 3 with the blocked cyclic paradigm, consisting of sets of four categorically related (arranged in rows) and heterogeneous (columns) contexts that vary according to the relative age dimension**.

**FIGURE 4:**
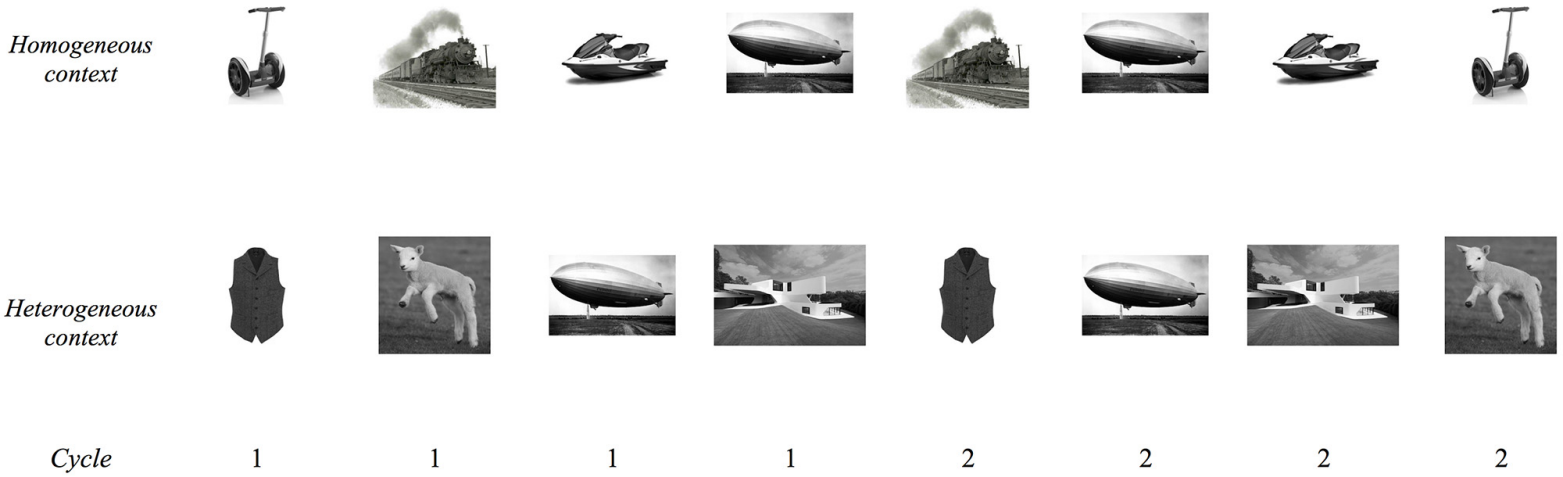
**An example sequence of trials over two consecutive cycles in the blocked cyclic paradigm employed in Experiments 2 and 3.** *Segway, train, jetski, airship* are from the homogeneous context (*vehicles* category), while *waistcoat, lamb, airship, house* are from a heterogeneous context.

Sixteen pictures (all gray-scale photographs) of familiar objects were selected from four semantic categories (*clothing, animals, vehicles,* and *buildings*) for the blocked cyclic paradigm. Half of these pictures consisted of relatively older/aged objects, while the other eight depicted relatively younger objects. Photographs were sourced from the internet. Pictures were arranged in a matrix of 4 × 4 items such that rows corresponded to categories and thus formed homogeneous sets of four items each, while columns formed the unrelated sets for the heterogeneous context (see **Figure 3**). Homogeneous and heterogeneous sets each comprised two older and two younger objects. Example series of consecutive trials in categorically homogeneous and heterogeneous sets are shown in **Figure 4**. Sixteen experimental lists were created using Mix software (Van Casteren and Davis, 2006). In each list, six presentation cycles were created for each homogeneous and each heterogeneous set, with pictures in each set presented in pseudorandom order in each cycle such that no consecutive items were the same. Homogeneous and heterogeneous blocks of 24 items were tested in alternation, with half of the participants beginning with the homogeneous context, in a Greco-Latin square design. The 192 item lists were split into two equal sessions of 96 trials.

The experiment was run with the Cogent 2000 toolbox (version 1.32^2^) in MATLAB (Version 7.13, R2011b, The Mathworks; Natick, MA, USA). Pictures (10 × 10 cm) were displayed centrally in gray-scale on a white background. Prior to the experiment proper, participants completed a block of practice trials in which they named all objects in random order, first with the correct basic-level name printed below and again without. Two sessions of 96 experimental items followed the practice block, with a brief rest break in between. On each trial, a fixation cross was presented for 500 ms, followed by the target picture for 1500 ms, then a blank screen for 1000 ms. RTs were recorded by a voice key implemented in Cogent 2000. Participants were instructed to name each picture as quickly and as accurately as possible. They were not informed about the relative age manipulation. After the experiment was completed, participants were presented with the 4 × 4 matrix of items and asked to categorize each as either older or younger. This served as a manipulation check for Experiment 3.

### Results

Technical errors (in which the voice-key failed to detect a response or was triggered by a non-speech sound) accounted for 1.3% of items being excluded. Trials with latencies deviating from each participant’s mean by 2.5 SD were labeled outliers and excluded (4.3%). Naming errors were infrequent (0.19%) and so were not subjected to analysis.

**FIGURE 5:**
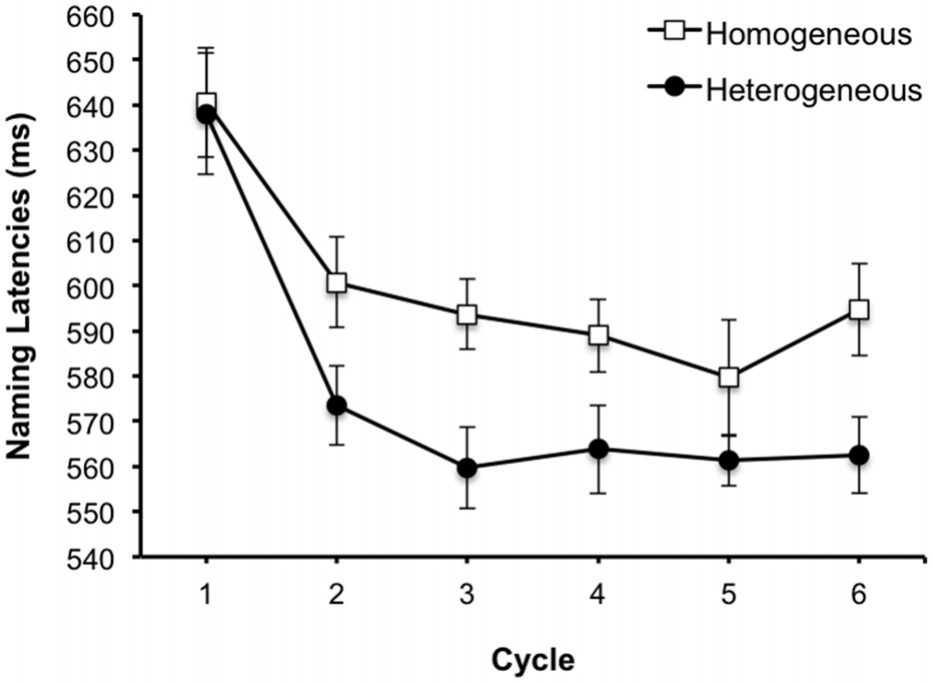
**Mean correct basic-level naming latencies as a function of context and cycle in the blocked cyclic paradigm.** Error bars represent 95% CIs calculated per Cousineau’s (2005) method.

We conducted a repeated measures analysis with semantic context and presentation cycle as within participants variables, and participants (*F*_1_) and items (*F*_2_) as random factors. The analysis revealed significant main effects of semantic context [*F*_1_(1,15) = 8.45, MSE = 3063.94, *p* = 0.011, $\eta_{p}^{2}$ = 0.36; *F*_2_(1,15) = 17.47, MSE = 1507.22, *p* < 0.001, $\eta_{p}^{2}$ = 0.54], such that RTs were slower overall for the homogeneous (*M* = 599.74 ms) compared to heterogeneous context (*M* = 576.51 ms) and presentation cycle [*F*_1_(5,75) = 45.04, MSE = 466.92, *p* < 0.001, $\eta_{p}^{2}$ = 0.75; *F*_2_ (5,75) = 40.92, MSE = 495.34, *p* < 0.001, $\eta_{p}^{2}$ = 0.73]. There was also a significant interaction by items [*F*_1_(5,75) = 2.10, MSE = 513.24, *p* = 0.075, $\eta_{p}^{2}$ = 0.123; *F*_2_(5,75) = 3.0, MSE = 419.15, *p* = 0.016, $\eta_{p}^{2}$ = 0.17]. As **Figure 5** shows, naming latencies become slower from the second cycle onward for the categorically homogeneous compared to heterogeneous sets, which is the typical pattern (see Belke and Stielow, 2013 for a review). A second ANOVA was conducted excluding data from the first cycle to determine if the interference effect in naming was cumulative over subsequent cycles (e.g., Oppenheim et al., 2010). This revealed significant main effects of semantic context [*F*_1_(1,15) = 13.39, MSE = 2239.81, *p* = 0.002, $\eta_{p}^{2}$ = 0.472; *F*_2_(1,15) = 21.60, MSE = 1439.66, *p* < 0.001, $\eta_{p}^{2}$ = 0.59] and presentation cycle [*F*_1_(4,60) = 3.48, MSE = 329.53, *p* = 0.013, $\eta_{p}^{2}$ = 0.188; *F*_2_(4,60) = 2.96, MSE = 403.77, *p* = 0.027, $\eta_{p}^{2}$ = 0.17]. However, the interaction was not significant (both *Fs* < 1.2, *p* > 0.05), nor was there any evidence of a linear trend to indicate a cumulative effect (both *Fs* < 1, *p* > 0.05). A paired *t*-test conducted on the means from the first presentation cycle was not significant (both *ts* < 1, *p* > 0.05).

The post-experiment manipulation check indicated that 98.83% of the participants’ responses were correct categorizations of the 16 pictures in **Figure 2** as older and young, thus confirming the transparency of the relative age manipulation.

### Discussion

A significant interference effect of semantic context was demonstrated on naming latencies using the novel set of categorically related object stimuli. Pictures named in categorically homogeneous sets were named more slowly than those in heterogeneous sets, and this effect emerged from the second presentation cycle onward, consistent with previous research. This effect did not accumulate over cycles (e.g., Belke and Stielow, 2013). Thus, we can be confident that our novel object stimuli induce the typical semantic context effect in the blocked cyclic paradigm with basic level naming despite varying in terms of relative age.

## Experiment 3: Blocked Cyclic Paradigm with Superordinate Categorisation

Given the typical semantic interference effect in basic level naming was observed in the blocked cyclic paradigm in Experiment 1, the aim of the following experiment was to determine whether a significant context effect could also be demonstrated with younger–older superordinate categorization using the identical stimuli in the blocked cyclic paradigm. The younger–older judgment preserves the semantic context manipulation, involves accessing conceptual features and eliminates a potential confound with identical living/non-living superordinate classifications in homogeneous contexts.

### Method

#### Participants

Sixteen students enrolled in a first year psychology course at the University of Queensland completed the experiment in exchange for partial course credit. None had taken part in any of the previous experiments. All participants reported normal or corrected-to-normal vision, and no history of neurological or psychiatric disorder, or substance dependence. All participants identified as right-handed, native English speakers. All participants gave written informed consent in accordance with the experimental protocol approved by the BSSERC of the University of Queensland.

#### Materials and Procedure

Materials (pictures and experimental lists) and procedure were identical to Experiment 2, with basic-level object naming replaced by the younger–older picture categorization task. Prior to the experiment proper, participants completed a block of familiarization trials in which they categorized all objects in random order, first with the correct category printed below and again without. For the two experimental sessions, participants were instructed to verbally categorize each picture as older or younger, as quickly and as accurately as possible.

### Results

Technical errors (in which the voice-key failed to detect a response or was triggered by a non-speech sound) accounted for 0.07% of items being excluded. Trials with latencies deviating from each participant’s mean by 2.5 SD were labeled outliers and excluded (3.2%). Naming errors were infrequent (0.26%) and not subjected to analysis.

**FIGURE 6:**
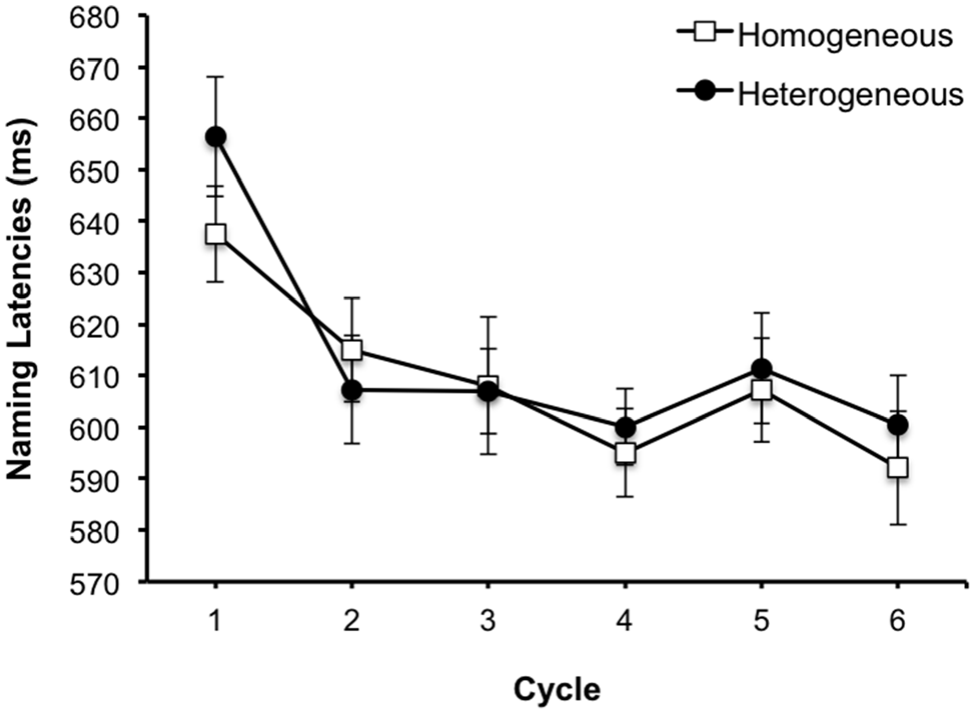
**Mean correct superordinate categorization latencies as a function of context and cycle in the blocked cyclic paradigm in Experiment 3.** Error bars represent 95% CIs calculated per Cousineau’s (2005) method.

The first set of analyses were identical to Experiment 2. We conducted a repeated measures ANOVA with semantic context and presentation cycle as within participant variables, and participants and items as random factors. The main effect of presentation cycle was significant [*F*_1_(5,75) = 23.57, MSE = 464.50, *p* < 0.001, $\eta_{p}^{2}$ *=* 0.61; *F*_2_(5,75) = 32.63, MSE = 341.48, *p* < 0.001, $\eta_{p}^{2}$ *=* 0.69]. However, neither the main effect of semantic context nor the interaction was significant (all *Fs* < 1.4, *p* > 0.05). As **Figure 6** shows, the mean categorization latencies became quicker following the first cycle. A second ANOVA excluding the first presentation cycle data again revealed a significant main effect of cycle [*F*_1_(4,60) = 3.32, MSE = 468.61, *p* < 0.05, $\eta_{p}^{2}$ = 0.18; *F*_2_(4,60) = 5.55, MSE = 259.51, *p* = 0.001, $\eta_{p}^{2}$ = 0.27]. However, the main effect of semantic context and interaction were not significant (all *Fs* < 1, *p* > 0.05). The linear trend was significant only by items [*F*_1_ < 2.3, *p* > 0.05; *F*_2_(1,15) = 8.09, MSE = 293.94, *p* = 0.012, $\eta_{p}^{2}$ = 0.35]. A paired *t*-test conducted on the first presentation cycle data was not significant [*t*_1_(15) = -1.87, *p* = 0.081; *t*_2_(15) = -2.02, *p* = 0.062].

We also conducted a mixed 2 (semantic context) × 6 (cycle) × 2 (basic level naming vs. semantic classification task) repeated measures ANOVA of the data from Experiments 2 and 3 with participants and items as random factors. Semantic context and cycle were varied within participants, while task/response type was varied between participants. This analysis revealed significant main effects of semantic context by items [*F_1_*(1,30) = 2.94, MSE = 2823.14, *p =* 0.097, $\eta_{p}^{2}$ = 0.09; *F*_2_(1,30) = 5.54, MSE = 1578.55, *p* = 0.025, $\eta_{p}^{2}$ = 0.16] and presentation cycle [*F*_1_(5,150) = 64.69, MSE = 465.71, *p* < 0.001, $\eta_{p}^{2}$ = 0.68; *F*_2_(5,150) = 71.29, MSE = 418.41, *p* < 0.001, $\eta_{p}^{2}$ = 0.70], and a significant interaction of context and cycle [*F*_1_(5,150) = 2.68, MSE = 542.19, *p* = 0.024, $\eta_{p}^{2}$ = 0.08; *F*_2_(5,150) = 3.79, MSE = 443.48, *p* = 0.003, $\eta_{p}^{2}$ = 0.11]. More importantly, there were also significant context × task [*F*_1_(5,150) = 6.6, MSE = 2823.14, *p* = 0.015, $\eta_{p}^{2}$ = 0.18; *F*_2_(5,150) = 11.71, MSE = 1578.55, *p* = 0.002, $\eta_{p}^{2}$ = 0.28] and cycle × task interactions [*F*_1_(5,150) = 3.97, MSE = 465.71, *p* = 0.002, $\eta_{p}^{2}$ = 0.12; *F*_2_(5,150) = 3.79, MSE = 418.41, *p* = 0.003, $\eta_{p}^{2}$ = 0.11]. The three-way interaction of context × cycle × task was not significant (both *Fs* < 1.2, *p* > 0.05).

A second mixed ANOVA excluding the first presentation cycle data likewise showed significant main effects of semantic context [*F*_1_(1,30) = 5.62, MSE = 2332.73, *p* = 0.024, $\eta_{p}^{2}$ = 0.16; *F*_2_(1,30) = 9.51, MSE = 1476.6, *p =* 0.004, $\eta_{p}^{2}$ = 0.24] and presentation cycle [*F*_1_(4,120) = 3.91, MSE = 399.07, *p* = 0.005, $\eta_{p}^{2}$ = 0.12; *F*_2_(4,120) = 4.85, MSE = 331.64, *p* = 0.001, $\eta_{p}^{2}$ = 0.14]. However, the interaction of context and cycle was not significant (both *Fs* < 1.2, *p* > 0.05). Again, the interactions of context × task [*F*_1_(1,30) = 7.29, MSE = 2332.73, *p* = 0.011, $\eta_{p}^{2}$ = 0.20; *F*_2_(1,30) = 11.6, MSE = 1476.6, *p* = 0.002, $\eta_{p}^{2}$ = 0.28] and cycle × task [*F*_1_(4,120) = 2.86, MSE = 399.07, *p =* 0.026, $\eta_{p}^{2}$ = 0.09; *F*_2_(4,120) = 3.1, MSE = 331.64, *p* = 0.018, $\eta_{p}^{2}$ = 0.09] were significant. The three-way interaction of context × cycle × task was not significant (both *Fs* < 1, *p* > 0.05). A mixed ANOVA on the first presentation cycle data failed to reveal a significant main effect of context [*F*_1_(1,30) = 0.96, MSE = 1125.68, *p* = 0.33, $\eta_{p}^{2}$ = 0.03; *F*_2_(1,30) = 1.63, MSE = 1635.9, *p* = 0.21, $\eta_{p}^{2}$ = 0.05] or context × task interaction [*F*_1_(1,30) = 1.63, MSE = 1125.68, *p* = 0.21, $\eta_{p}^{2}$ = 0.05; *F*_2_(1,30) = 2.07, MSE = 1635.9, *p* = 0.16, $\eta_{p}^{2}$ = 0.07].

We conducted one final analysis on the data from Experiment 3 to establish the likelihood of the null effect obtained for the classification task in the original 2 × 6 ANOVA. This involved calculating a Bayes Factor (Rouder et al., 2012) for the main effect of context using JASP software (Love et al., 2015). For the by-participants and by-items analyses, the Bayes factors B_10_ were 0.256 and 0.255, respectively. Thus, the data are ∼4 times more likely to have occurred under the null than for the alternative hypothesis. According to Jeffreys (1961), this constitutes ‘substantial’ evidence for the null hypothesis.

### Discussion

We observed a significant interaction between semantic context and the nature of the task employed in the blocked cyclic paradigm in Experiments 2 and 3. Although a significant interference effect was observed for basic level naming in Experiment 2, we failed to observe a significant effect of semantic context on younger–older superordinate categorization in Experiment 3. This latter result is consistent with Damian et al.’s (2001) findings with orientation judgments.

## General Discussion

In Experiment 1, we found a facilitative effect of categorically related contexts on superordinate (living/non-living) verbal categorization in the continuous paradigm. This effect was also cumulative over ordinal positions. Experiment 2 showed that the typical interference effect of a categorically related context on basic-level naming in the blocked cyclic paradigm could be induced using stimuli that also varied in terms of the core semantic dimension of relative age. Experiment 3 failed to demonstrate effects of categorically related contexts with the identical object stimuli and blocked cyclic procedure from Experiment 2 when the task was changed to superordinate (younger–older) verbal categorization. These findings indicate that the semantic context effects in the continuous and blocked cyclic paradigms are likely to have different origins.

In Experiment 1, we replicated Belke’s (2013) finding of cumulative facilitation in the continuous paradigm with manual living/non-living judgments, without the modification of a picture familiarization phase. Semantic context effects in superordinate categorization are attributed to the activation of shared conceptual features converging on the *same* response, resulting in facilitation, rather than spreading to multiple lexical candidates, as is proposed in the case of basic level naming (Lupker and Katz, 1981; Glaser and Düngelhoff, 1984; Glaser and Glaser, 1989; Humphreys et al., 1995; Costa et al., 2003; Hantsch et al., 2012). This result therefore supports proposals that the semantic context effect in the continuous paradigm arises at the level of conceptual representations (e.g., Belke, 2013) or in conceptual-to-lexical connections (Howard et al., 2006; Oppenheim et al., 2010). Although devised to simulate findings with basic-level naming, both the Howard et al. (2006) and Oppenheim et al. (2010) computational models could be modified to accommodate the superordinate categorization results (see Belke, 2013 for a discussion of the relevant modifications). Similarly, Belke’s (2013) conceptual feature accumulation hypothesis could be augmented by an incremental learning mechanism to account for the long-lasting nature of the context effect (e.g., Damian and Als, 2005; Howard et al., 2006).

We failed to find evidence of a context effect with younger–older judgments in the blocked cyclic paradigm (Experiment 3), despite demonstrating the typical semantic interference effect with the identical stimuli and procedure when the task was basic-level naming (Experiment 2). Taken together, these findings are consistent with Damian et al.’s (2001) finding with orientation judgments, and support a lexical rather than conceptual origin for context effects in this paradigm. Objects varying along a younger–older dimension can be found in almost all categories, and the relative age distinction is a core semantic adjectival class across languages. Although consistent with Damian et al.’s (2001) finding with orientation judgments, the failure to observe a significant facilitation effect for categorically related contexts with younger–older judgments contrasts with Belke’s (2013) recent findings with living/non-living judgments in a modified blocked cyclic paradigm. Below we consider three possible reasons for the discrepant results across studies using classification tasks: (1) the use of verbal vs. manual responding, (2) the type of superordinate category judgment, and (3) the introduction of a lag modification.

A possible explanation might be our use of verbal responding for the superordinate categorization task, as Belke (2013) employed a manual response. For example, it might be the case that a manual response is more sensitive than a verbal response. However, Damian et al. (2001) employed manual responses for their orientation judgments and obtained results consistent with ours in the blocking paradigm. Further, we observed a significant semantic context effect in the continuous paradigm in Experiment 1 with verbal living/non-living responses that was consistent with Belke’s (2013) finding using manual living/non-living responses in the same paradigm. Thus, response modality does not appear to be the reason for the different results observed across studies with superordinate categorization in the blocked cyclic paradigm. Of note, in the speech production literature, verbal superordinate categorization has been compared more frequently with basic-level naming because it has the advantage of involving the identical output channel (Lupker and Katz, 1981; Glaser and Düngelhoff, 1984; Glaser and Glaser, 1989; Humphreys et al., 1995; Costa et al., 2003; Hantsch et al., 2012).

Another possibility is that both orientation and younger–older judgments do not involve accessing the *same* conceptual features as living/non-living judgments, and so feature overlap is less relevant for performing the former tasks. However, in two multi-experiment studies Boucart and Humphreys (1997) were able to demonstrate orientation judgments *do* involve conceptual processing via context manipulations with categorically related objects. Semantic congruity effects have also been observed with younger–older judgments, reflecting processing of conceptual features around the midpoint of a context-dependent scale (e.g., Ellis, 1972; Ryalls and Smith, 2000). Recognizing a *mammoth*, for example, will involve accessing features common to the category of animals (and *elephants* in particular), in addition to distinguishing features (e.g., *fur*) to support both a ‘living’ and an ‘old’ decision. All modern theories of conceptual organization assume that meaning computation involves access to both shared and distinguishing features (Vigliocco et al., 2004; McRae et al., 2005; Vieth et al., 2014). Thus, it seems unlikely that the type of superordinate comparative judgment could be responsible for the different findings.

We consider the most plausible explanation for the discrepancy in findings is that modifying the procedure by including a lag manipulation alters the nature of the mechanisms operating in the conventional blocked cyclic paradigm by removing an obvious task set (e.g., Belke and Stielow, 2013). Damian and Als (2005; also Navarrete et al., 2012; Belke, 2013) observed that modifying the blocking paradigm with intervening, unrelated items resulted in the context effect manifesting from the first rather than second cycle. Given the above, we interpret our findings from Experiment 3 as supporting Damian et al.’s (2001) interpretation that the semantic interference effect in the conventional blocked cyclic paradigm both arises and has its locus in lexical-level processing. The absence of a context effect with younger–older classification is not consistent with proposals for an origin in conceptual-level processing or in conceptual-to-lexical connections (cf. Belke, 2013; Oppenheim et al., 2010). Thus, the semantic interference effects in the continuous and conventional blocked cyclic paradigms likely have different origins, as we elaborate below. However, our results are not able to adjudicate between accounts proposing competitive vs. non-competitive lexical selection mechanisms (Damian et al., 2001; Howard et al., 2006; Oppenheim et al., 2010).

If the context effect in blocked cyclic naming arose at the conceptual level, then one might expect other types of semantic relations to show similar context effects. For example, Abdel Rahman and Melinger (2007) reported an interference effect with associative relations in basic level naming. However, other studies have not replicated this finding (e.g., de Zubicaray et al., 2014). Interestingly, Navarrete et al. (2014) have recently proposed that the slowing of naming latencies in the blocked cyclic paradigm does *not* reflect a semantic interference effect. According to their account, the effect is due to less repetition priming in the categorically related compared to unrelated contexts. Thus, what appears to be “semantic interference” is instead a relative speeding of naming responses in the unrelated condition. However, it is worth noting that Navarrete et al. (2014) employed a highly modified blocking paradigm to support their proposal.

The failure to observe a context effect with either younger–older or orientation judgments could also be considered consistent with the assumption that the context effect in blocked cyclic naming does not reflect semantic interference, if semantic interference is proposed to necessarily originate in conceptual processing or in conceptual-lexical links (e.g., Belke, 2013). Instead, the encoding of a set of task relevant representations in working memory might be the crucial difference between paradigms as Belke and Stielow (2013) suggested, because it potentially implicates another mechanism, i.e., *proactive interference* (PI; e.g., Crowther and Martin, 2014). In the classic *buildup of PI* procedure (see Wickens, 1970), participants recall word triads that they have been asked to study. Recall of each triad is examined as a function of its serial position in a sequence of trials presenting triads from the same category. While the initial triad is typically recalled well, performance declines for subsequent related triads. Later studies showed that buildup of PI is also reflected in response latencies (e.g., Rohrer and Wixted, 1994). Thus, encoding the categorically related set in the first cycle might contribute to a buildup of PI in working memory that manifests as a context effect in naming latencies from the second cycle onward.

The classical view of the buildup of PI is that it arises during response selection, resulting from competition between target and non-target information at retrieval (e.g., Postman and Underwood, 1973). This corresponds to the stage of lexical selection in models of word production (e.g., Levelt et al., 1999), and so is consistent with Damian et al.’s (2001) original proposal concerning the origin and locus of the context effect in blocked cyclic naming. A buildup of PI account is also consistent with the correlations observed between working memory span and the context effect in the blocked cyclic paradigm (e.g., Belke, 2008; Crowther and Martin, 2014), as PI and span are closely related (e.g., Lustig et al., 2001)^3^. The account can also explain the absence of a context effect with younger–older or orientation judgments: if the task sets are encoded in terms of category membership, then this is likely to produce response competition during name retrieval in the homogeneous context. However, this is unlikely to produce competition in judgments based on dimensional adjectives such as relative age or typical orientation as they cross the boundaries of taxonomic categories (e.g., Wickens et al., 1976).

As our primary aim in Experiment 1 was to replicate Belke’s (2013) finding of a cumulative facilitation effect in continuous naming with living/non-living judgments, we did not employ younger–older judgments as per Experiment 3. Thus, a limitation of the present study is that we were unable to directly compare continuous and blocked cyclic naming paradigms using the same type of semantic classification. The majority of the items in the categories employed by Howard et al. (2006) do not differ on the relative age dimension. While it might be possible to create a version of the continuous paradigm for use with younger–older judgments, this would necessitate employing fewer categories.

More broadly, the debate concerning the conceptual vs. lexical origins of semantic interference effects in naming paradigms parallels the debate about similar context effects in visual search paradigms. In the latter paradigms, participants are required to select a pre-specified target from an array of semantically related objects via a button-press corresponding to the spatial arrangement of the objects. Results from these paradigms are interpreted typically in terms of relatedness effects in visual search inducing competition in the allocation of visual attention (Meyer et al., 2007; Belke et al., 2008). However, there has been some debate about whether lexical representations become activated in these search paradigms and influence competition with the target for selection. As the paradigm does not explicitly involve name retrieval, some authors have argued that the semantic interference effect must therefore arise at a pre-lexical, conceptual level (Jefferies et al., 2007; Campanella and Shallice, 2011). Yet, similar context effects have been observed with phonologically related objects in visual search, indicating object names can become activated automatically even in tasks that do not require explicit lexical retrieval, and result in response competition (Meyer et al., 2007; Görges et al., 2013).

Memory mechanisms also appear involved in the semantic interference effect in the visual search paradigm (Belke et al., 2008; Balani et al., 2010; Hout and Goldinger, 2010). For example, concurrent performance of a digit retention task exacerbates the semantic interference effect, as does presenting trials in a blocked cyclic procedure (Jefferies et al., 2007; Belke et al., 2008; Gardner et al., 2012). In addition, evidence for an additive effect of working memory and semantic context in visual search has been interpreted as indicating participants are able to establish task-relevant vs. irrelevant representations to guide performance (Balani et al., 2010; Hout and Goldinger, 2010), analogous to proposals for the blocked cyclic naming paradigm (Belke and Stielow, 2013).

Clearly, experimental paradigms that manipulate semantic contexts and task-relevant representations in memory are complicated. The continuous paradigm might therefore be more suitable for exploring processes involved in conceptual-lexical access in spoken word production, while the blocked cyclic paradigm might be more suitable for exploring interactions between lexical retrieval and memory-related processes, due to its establishing a task set in memory from the first cycle onward. For example, Crowther and Martin (2014) argued that if participants were using lexical-semantic representations in working memory to perform the blocked cyclic naming paradigm, then this reflected a task-specific process rather than a mechanism involved in word production in more naturalistic settings. This task-specific process would therefore need to be added to existing models of word production to explain semantic context effects in the blocked cyclic paradigm.

## Conflict of Interest Statement

The authors declare that the research was conducted in the absence of any commercial or financial relationships that could be construed as a potential conflict of interest.

## Footnotes

1 www.vislab.ucl.ac.uk/cogent_2000.php

2 www.vislab.ucl.ac.uk/cogent_2000.php

3 Crowther and Martin (2014) failed to observe a positive correlation between the context effect in the blocked cyclic naming paradigm and a measure of PI from the recent-probes task (Monsell, 1978). However, the recent-probes task provides a measure of interference due to temporal recency in recognition memory, which is different to the buildup of PI due to semantic context in free recall. There is increasing evidence that the maintenance of semantic representations in working memory involves a distinct capacity (e.g., Majerus et al., 2004; Shivde and Anderson, 2011; Nishiyama, 2014).
